# Knowledge, perceptions, and consumption behaviour of cosmetics among undergraduates of Sri Lanka: a descriptive cross-sectional study

**DOI:** 10.3389/fpubh.2023.1184398

**Published:** 2024-01-05

**Authors:** Lahiru Udayanga, Nirma Subashini, Menuka Udugama, Prabha Silva, Tharaka Ranathunge

**Affiliations:** ^1^Department of Biosystems Engineering, Faculty of Agriculture and Plantation Management, Wayamba University of Sri Lanka, Makadura, Sri Lanka; ^2^Department of Horticulture and Landscape Gardening, Faculty of Agriculture and Plantation Management, Wayamba University of Sri Lanka, Kuliyapitiya, Sri Lanka; ^3^Department of Agribusiness Management, Faculty of Agriculture and Plantation Management, Wayamba University of Sri Lanka, Kuliyapitiya, Sri Lanka; ^4^Department of Zoology, Faculty of Science, Eastern University, Chenkaladi, Sri Lanka

**Keywords:** knowledge attitudes and practices, socio-economic, cosmetic consumption, Sri Lanka, undergraduates

## Abstract

**Background:**

Despite the increasing usage of personal care products among young adolescents in Sri Lanka, limited studies have been conducted to understand the behaviour of cosmetic users and associated health complications. Therefore, the current study was conducted to evaluate the cosmetic consumption related behaviour of the undergraduate community in Sri Lanka and to identify the driving factors behind the incidences of cosmetic related adverse health effects.

**Method:**

An analytical cross-sectional study was conducted by recruiting 421 undergraduates from five state universities in Sri Lanka through stratified random sampling, as the study population. Information on socio-demographic factors and cosmetic consumption behaviour of the participants were acquired through a self-administrated structured questionnaire, along with Knowledge, Attitudes and Practices (KAP) relevant for cosmetic usage. The Binary Logistic Regression model was used to determine the significant socio-demographic driving factors on cosmetic usage among the undergraduate community in Sri Lanka at a confidence level of 95%.

**Results:**

Around 96.4% of the study population used one or more cosmetic products (77%) out of which, 75.3% experienced cosmetic related adverse health effects. Skin dryness (24%), acne (21%), allergies (20.5%) and rashes (19.8%), were identified as the most dominant adverse health effects, related to cosmetic usage. Perfumes (65.6%), face cream (63.2%) and body lotion/hand cream (60.6%), were the mostly used cosmetic products. Even though half of the study population exhibited higher levels of good practices during purchase (54.9%) and application (52%) of cosmetics, around 47.5% were characterized with a low level of knowledge on cosmetics. Gender, academic year, knowledge on cosmetics, monthly expenditure on cosmetics, source of recommendation for cosmetics, practices related to purchase and consumption of cosmetics and preference to receive medical care in case of cosmetic related emergency were recognized as significant risk factors (*p* < 0.05) associated with the incidence of cosmetic related adverse health effects among undergraduate students.

**Conclusion:**

Given the high prevalence of cosmetic related adverse health effects, the health authorities of Sri Lanka should pay more attention towards the wellbeing and responsible cosmetic usage among undergraduates. Designing of effective tools and regulations to monitor the cosmetic market and improving the knowledge on cosmetics are recommended to ensure safe cosmetic usage within the country in general and of adolescent users in particular.

## Introduction

Articles used for beautification, cleansing, or altering physical appearance, could be identified as cosmetics, which include a variety of products ranging from make-up, skin care products and perfumes to contact lenses available in various forms (1). Cosmetic products can be natural, synthetic or a mixture of both. With unrealistic beauty standards set mainly by the media, use of cosmetic products to enhance beauty has intensified more than ever, world-wide including South Asia (2). Proper use of cosmetics could aid to maintain youthfulness, health, aesthetics, and well- being, especially with skin or hair care products. Given this, irrespective of country or culture, the cosmetics market grows rapidly and changes frequently due to consumers who highly value physical appearance and quality of life (3).

The Cosmetic market in Sri Lanka tends to play a huge role in defining beauty and building up self confidence in users, causing the industry to become extremely competitive. Cosmetic products are increasingly being used by Sri Lankans, for numerous reasons ranging from improving appearance to camouflaging skin conditions (4, 5). The younger generation today, although still carrying the affinity to traditional beauty standards such as preference for fairer skin, are also much more knowledgeable and inquisitive. The cosmetic market is ready to fill the gaps with new brands and product lines (5). The Surge of new cosmetic trends globally and regionally has a great influence on the local users. Thus, more young users are moving towards whitening and anti-aging products to define beauty and maintain themselves in good conditions (2, 4).

Literature shows that certain compounds in these products can have short term as well as long term adverse health effects that could range from skin irritations and rashes to long term conditions such as loss of skin elasticity or infections that may be life threatening (4). Several studies have highlighted that make-up could be absorbed through micropores into the body, leading to numerous infections and inflammations (6). Skin whitening through bleaching and other means, which is another prevalent trend in South Asia, has been found to reduce physiological pigmentation of the skin (7). A study carried out with female university students in India has revealed that lack of information and knowledge about cosmetics and their ingredients could lead to long term impacts such as infections or skin conditions, which are not yet assessed (8). A high number of studies have reported the presence of hydroquinone, glutathione, topical steroids, ammonia, hydrogen peroxide and heavy metals in skin whitening products, which could lead to adverse long-term impacts (9–11). Therefore, awareness on purchase of products with safe ingredients and proper usage patterns, plays a critical role in using cosmetics without any adverse health impacts.

Apart from products, beauty salons could also be places contaminated with microbes making it possible to contract infections during the application of makeup and other related products (12). However, despite the potential threats and risks, users still purchase and apply cosmetic products and visit salons as appearance, image and self-confidence are strongly associated with use of these products, even with certain temporary discomforts (13–15). According to the findings of Jagadeeshan et al. (16), around 60% of participants who underwent skin whitening experiences have had adverse effects such as skin irritation. Interestingly, around 90% of this fraction had undergone the above procedure, despite of believing that bleaching is not safe. Such studies clearly depict that the short-term benefits such as appearance could outweigh the perceived negative impacts of using cosmetics.

A considerably high fraction of the local communities in Sri Lanka are constant appliers of any kind of cosmetics and majority of the users are not aware of the effects of the frequent use of cosmetics; makeup and personal care products (4). A recent study conducted with Saudi female students has revealed that majority of the students are unaware of the harmful effects or skin problems related to the constant use of cosmetics (17). A previous study conducted in Colombo, Sri Lanka revealed that around 90% of the study population use at least one cosmetic product (4). Unlike in the past, males also show an affinity towards using cosmetics at present. French and Canadian men have been found to purchase grooming products at notable levels, due to different motivations and driving factors such as self-image, aging effects, state of health, and physical attractiveness (18). Owing to this high tendency of using cosmetics, it is as well necessary to identify the level of awareness of the consumers on the harmful effects of routine applications of the cosmetics.

Studies show that knowledge and attitudes play dominant roles on purchasing behaviour of users (19, 20). A variety of socio-economic factors such as level of education, monthly income, ethnicity, nature of the life-style and social background as well product attributes and marketing have been found to influence the purchasing behaviour and use of cosmetics (19, 21–23). A cohort of University students is a unique sample given their education and age as they have a tendency of long-term continuous use of cosmetics. Many studies that focused on the cosmetic consumption behaviour and attitudes of young population have reported poor awareness on cosmetic usage, with high prevalence of mild and moderate side effects of cosmetics (21, 24).

Therefore, shaping attitudes of adolescents, while giving them a sense of empowerment has been identified as an important factor in promotion of safe cosmetic consumption (25). Despite the growing market and purchase of cosmetic products in South Asia in general and Sri Lanka (4, 5) in particular, the behaviour of cosmetic users has not been studied comprehensively. Therefore, the current study was conducted to evaluate the knowledge, attitudes/perceptions and behaviours/practices on cosmetic consumption and to characterize the driving factors influencing cosmetic usage among adolescent undergraduates in the university system of Sri Lanka. The findings of this study will be immensely important to fill the knowledge gaps related to the cosmetic consumption behaviour among adolescents in Sri Lanka, while characterizing their knowledge and attitudes on safe cosmetic consumption. In addition, outcomes of this study will provide insights to strengthen the current monitoring and regulatory mechanisms and promote responsible cosmetic usage, particularly in an Asian perspective.

## Methodology

### Selection of study population

An analytical cross-sectional survey was conducted from January to May 2021. The ethical approval for this study was acquired from the Wayamba University Ethical Review Committee. The Lwanga and Lemeshow equation (26) was used to calculate the required sample size, as 384 undergraduates. Precision was maintained as 5% and the critical value of specified confidence level (95%) was used as 1.96, while the population proportion was set as 0.5 (50%). Subsequently, the sample size was increased up to 421 undergraduates, expecting a dropout rate of 10%.

As the study population, a total of 421 randomly selected undergraduates were recruited from five state universities in Sri Lanka, namely University of Colombo, University of Moratuwa, University of Kelaniya, South-Eastern University of Sri Lanka, Wayamba University of Sri Lanka. Stratified random sampling technique was used to recruit undergraduates for the current study, while the academic discipline (technology, agriculture, biological sciences and commerce and management) and academic year (first year to final year), were considered as strata. The selection of students was done in such a way to ensure effective representation of different academic disciplines and levels of undergraduates. Being an undergraduate student of aforementioned universities in Sri Lanka and above 18 years of age were considered as the inclusion criteria. Undergraduates who were not in a condition to answer the questions or who refused to participate the survey were excluded from the study.

### Data collection

Socio-economic characteristics, Knowledge, Attitudes and Practices (KAPs) of the selected undergraduates on cosmetic purchasing and consumption were collected using a pre-tested structured self-administered questionnaire, prepared in three local languages (Sinhala, English and Tamil). The questionnaire was developed to suit the local conditions, based on a comprehensive literature survey. The content validity of the questionnaire was assessed by a panel of 10 experts, comprised of academics and medical officers. The Confirmatory Factor Analysis (CFA) was conducted to test construct/scale reliability, unidirectionality and construct validity. The Cronbach’s alpha coefficients obtained for the questionnaire indicated that the questionnaire and tools used in the study had good internal consistency. The questionnaire was pre-tested with *n* = 25 students selected proportionately from the population. The validated and revised questionnaire was used for the actual survey.

The questionnaire covered four main areas as, 1. Socio-demographic information (age, gender, nature of the residing locality, ethnicity, monthly income, field of study and the level of study of the undergraduates); 2. Cosmetic consumption behaviour (types of cosmetics used, frequency of use, monthly expenditure on cosmetics, practices on purchase and use of cosmetics); 3. Knowledge on cosmetics (knowledge on cosmetic use, harmful ingredients and the possible risks); and (4) attitudes towards cosmetic consumption (reasons for cosmetic usage, considerations in purchasing cosmetics and perceived risks of cosmetic use).

### Data interpretation and statistical analysis

All collected data were double-checked and verified on the same day for completeness and consistency prior entering into Microsoft Access^®^ data sheets (Version, 2016). Quality control procedures were followed throughout the process by trained personnel, while cross tabulations and logical checks were conducted to maintain the accuracy of data. Discrepant data were checked against original data forms and any mistakes were promptly corrected. The Binary Logistic Regression (BLR) was used to calculate the Odds Ratio (OR) and the 95% Confidence Intervals of the OR for socio-demographic driving factors behind the incidence of cosmetic related adverse health effects. As knowledge, attitudes and perceptions are latent factors, in order to quantify, statements reflecting these factors were provided to respondents on a five-point Likert scale. All statistical analysis were done using the SPSS (package 23).

## Results

### Demographic and socio-economic factors

Majority of the respondents were females (78.4%). Around, 55.1% of them belonged to the age group of 20 to 23 years, followed by 24-to-26-year age group (41.3%). Sinhala (71.3%), Muslim (17.6%) and Tamil (10.7%) were the major ethnicities of the respondents, while majority were residing in semi–urban (51.1%) and rural (29.7%) settings (Table 1). Respondents of the study belonged to diverse academic fields of study, where majority studied in technology stream (36.6%), followed by agriculture (20.2%), biological sciences (19.7%) and commerce and management (11.2%). First year undergraduates dominated within the study population (32.3%), while fourth year (final year) undergraduates denoted the least frequency of 17.8% (Table 1). Total monthly income of relatively higher fraction of respondents was below LKR 25,000 (31.4%), followed by LKR 50,001 to 75,000 (27.1%). Meanwhile, only 20% of the families were receiving a monthly income above LKR 75,000 (Table 1).

**Table 1:** Socio-demographic factors of the study population.

Parameter		Respondents	
		*n*	%
Gender	Male	91	21.6
	Female	330	78.4
Age (years)	20–23	232	55.1
	24–26	174	41.3
	>26	15	3.6
Ethnicity	Sinhala	300	71.3
	Tamil	45	10.7
	Muslim	74	17.6
	Burgher	1	0.2
	Other	1	0.2
Nature of the residing locality	Rural	125	29.7
	Semi–urban	217	51.1
	Urban	79	18.8
Academic field of study	Agricultural and food sciences	85	20.2
	Arts & social sciences	15	3.6
	Biological sciences	83	19.7
	Commerce and management	47	11.2
	Engineering	3	0.7
	Mathematical and physical sciences	29	6.9
	Medical science	5	1.2
	Technology	154	36.6
Academic year	First year	136	32.3
	Second year	104	24.7
	Third year	106	25.2
	Fourth year	75	17.8
Total family income	<25,000	132	31.4
	25,001 – 50,000	91	21.6
	50,001 – 75,000	114	27.1
	75,001 – 100,000	60	14.3
	>100,000	24	5.7

*n*, number of respondents in the sample.

### Consumption behaviour and usage patterns of cosmetics

Almost the entire study population (96.4%), were using one or more cosmetic or personal care products. Perfumes (65.6%), face cream (63.2%) and body lotion/hand cream (60.6%), were the mostly used cosmetic products by undergraduates, while hair dye (3.1%), anti-aging products (2.1%) and complexion powder (8.3%) were consumed least, as shown in Table 2. Around 59.4% of the respondents were spending <LKR 1000 on cosmetics, while another 30.4% spent LKR 1000 to 2,500 per month. Interestingly, only 10.3% were spending > LKR 2500 for cosmetics during a month. Majority of the undergraduates (77.0%) were using 1 to 2 cosmetic products at once, while 19.2% were using 3 to 4 products. These cosmetic products were often consumed at a frequency of once a day (42.3%) and twice a day (32.8%). Enhancing the appearance (89.3%), improving self- image/ self-esteem stimulation (89.3%), being fashionable (67.9%) and protecting the skin against external environment (62.0%) were the prominent reasons for using cosmetics (Table 2).

**Table 2:** Consumption behaviour and usage patterns of cosmetics.

Parameter		Respondents	
		*n*	%
Type of cosmetic items used	Face cream	266	63.2
	Body lotion/hand cream	255	60.6
	Sunscreen	55	13.1
	Nail polish	117	27.8
	Eye makeup	62	14.7
	Perfumes	276	65.6
	Deodorants / Deo-spray	95	22.6
	Lipsticks	84	20.0
	Complexion powder	35	8.3
	Hair dye	13	3.1
	Anti-aging	9	2.1
Monthly expenditure for cosmetics (LKR)	<1,000	250	59.4
	1,000–2,500	128	30.4
	2,500–5,000	20	4.8
	>5,000	23	5.5
Number of cosmetic items used per day	1 to 2	324	77.0
	3 to 4	81	19.2
	5 to 6	8	1.9
	>6	3	0.7
Frequency of using cosmetics	Once a day (either morning or night)	178	42.3
	Night time only	24	5.7
	Twice a day	138	32.8
	Once a week	37	8.8
	2–3 days per week	44	10.5
Reasons for using cosmetic products	To enhance good looks/appearance	376	89.3
	Improve self- image/ Self-esteem stimulation	368	87.4
	Being fashionable/ Enhance style	286	67.9
	To cover up scratches/ wounds/marks	171	40.6
	Personal hygiene	225	53.4
	Medical conditions	146	34.7
	Create a unique image	190	45.1
	Protection against external environment	261	62.0
	Delay the process of aging/ keep young	145	34.4
	Because of friends	187	44.4
	For enjoyment	151	35.9
Purchasing source of cosmetics	Cosmetic shops	260	30.4
	Cosmetic agents	24	2.8
	Pharmacy	228	26.7
	Local shops	93	10.9
	Supermarkets	171	20.0
	Borrow from friends	19	2.2
Source of recommendation	Doctors	44	10.5
	Beauticians/ Pharmacists	100	23.8
	Friends	164	39.0
	Parents	53	12.6
	Elder Siblings	37	8.8
	Fiancé/Fiancée/Spouse	23	5.5
Have you ever experienced any adverse effects of cosmetic products?	Yes	317	75.3
	No	104	24.7
Types of adverse effects	Skin dryness	194	24.0
	Rash	160	19.8
	Allergy	166	20.5
	Acne	170	21.0
	Eye irritation	38	4.7
	Dermatitis	24	3.0
	Alopecia/Loss of hair	58	7.2
	Other	53	6.5
What did you do, when you experienced such adverse effects?	Medical consultation	108	25.7
	Stopped use of the suspected cosmetic product	191	45.4
	Permanently stopped using any cosmetic products	25	5.9
	Shifted the brand	38	9.0
	Inform friends and others about the issue	32	7.6
	Complaint	9	2.1
	Stopped use of the products only until the symptoms are gone	18	4.3

*n*, number of respondents in the sample; LKR, Sri Lankan rupees.

Cosmetic shops (30.4%), pharmacies (26.7%) and supermarkets (20.0%) were the mostly preferred places for purchasing cosmetic products. Meanwhile, a relatively lower fraction of respondents was borrowing cosmetic products from friends (2.2%) or purchasing through cosmetic agents (2.8%), as shown in Table 2. Friends (39.0%) were found to be the dominant source of information for purchase of cosmetic products, followed by beauticians/ pharmacists (23.8%). Interestingly, only 10.5% of the undergraduates were seeking the advice of doctors in selecting cosmetic products for them. Around 75.3% of the participants, had experienced different adverse effects of using cosmetic products, such as skin dryness (24.0%), acne (21%), allergies (20.5%) and rashes (19.8%). Meanwhile, a relatively lower faction of respondents (6.5%) had experienced several other issues such as changes skin colour, skin wounds, skin pigmentation and white heads. Majority of the undergraduates had stopped using the suspected cosmetic products (45.4%), after experiencing such issues, while only 25.7% had pursued medical attention. Meanwhile, a notable faction had shifted the brand of cosmetics (9%), while only 2.1% had complained to relevant authorities.

### Practices related to purchase and consumption of cosmetics

Available ingredients (75.5%), easy applicability (68.4%), brand (68.7%), medical recommendations (65.5%) and approval by the professionals/experts (62.5%) acquired by cosmetic products were perceived as critical considerations in purchasing cosmetic products by undergraduates. However, the studied undergraduate population was paying a limited attention on the packaging and advertising of the products (Table 3). Majority of the respondents were always paying attention to the expiry and manufacture dates (56.5%), usage instructions (45.4%) and the ingredients (40.4%) during purchase of cosmetic products. Meanwhile, a notable fraction was often check for manufacturing company (47.5%), brand credibility (41.1%) and quality certifications (40.1%). However, relatively lower fraction of respondents was inspecting the price and production license of the company, as shown in Table 4.

**Table 3:** Characteristics of cosmetic products considered during purchase.

Practice	Degree of Importance (%)				
	Least important	Low importance	Neutral	Important	Highly important
Brand	5.5	3.1	22.8	40.4	28.3
Fragrance of the product	4.3	6.7	28.0	46.3	14.7
Ingredients Herbal/ natural	3.6	2.4	18.5	39.4	36.1
Easy applicability	4.0	3.8	23.8	48.9	19.5
Experts approval/testing	2.6	7.1	29.2	39.7	22.8
Packaging	5.9	11.9	36.1	36.6	9.5
Advertising	10.2	18.3	41.6	25.4	4.5
Certification	4.5	7.1	21.4	39.7	27.3
Medical advice	4.0	6.4	24.0	34.4	31.1
Price	4.0	4.3	28.3	38.2	25.2

**Table 4:** Practices during purchase and use of cosmetic products.

Practice	Frequency of practice (%)				
	Very rarely	Rarely	Sometimes	Often	Always
Practices during purchase of cosmetic products					
Check for manufacturer company	3.3	2.0	18.5	47.5	28.7
Read expiry and manufacture date	3.1	1.2	8.8	30.4	56.5
Always read label for ingredients	3.3	1.4	17.1	37.8	40.4
Always read the label for consumption data	3.3	2.8	13.1	35.4	45.4
Check for production license	3.1	4.6	31.8	35.9	24.7
Check for quality certifications	2.6	2.6	23.5	40.1	31.1
Check Brand credibility	2.9	4.5	23.0	41.1	28.5
Check for the original brand logo and place of manufacture	3.1	3.3	28.0	36.3	29.2
Check for the price, rather than other factors	5.2	7.4	27.6	38	21.9
Practices during consumption of cosmetic products					
Daily wash off the applied products	2.6	4.5	20.9	40.1	31.8
Prevent contamination of the products	1.4	3.8	27.8	43.9	23.0
Perform patch /sensitivity test before using	1.9	5.2	33.0	40.4	19.5
Properly store the products as indicated on the label of the product	1.7	4.3	31.8	42.0	20.2
Mix two or more products	23.8	33.3	27.3	12.1	3.6
Shared cosmetic products with others	11.9	17.6	38.2	25.7	6.7
Use traditional cosmetics	5.0	7.8	31.4	38.2	17.6
Use homemade cosmetic products over market products	5.9	9.5	31.8	31.8	20.9
Avoid cheap lotions, thinking that they have a low quality	5.9	12.8	35.2	29.9	16.2
Specify products that you need before purchase	1.9	6.4	32.5	42.3	16.9
Go for available alternatives in case of unavailability of required product	5.7	12.1	38.5	34.2	9.5

**Figure 1:**
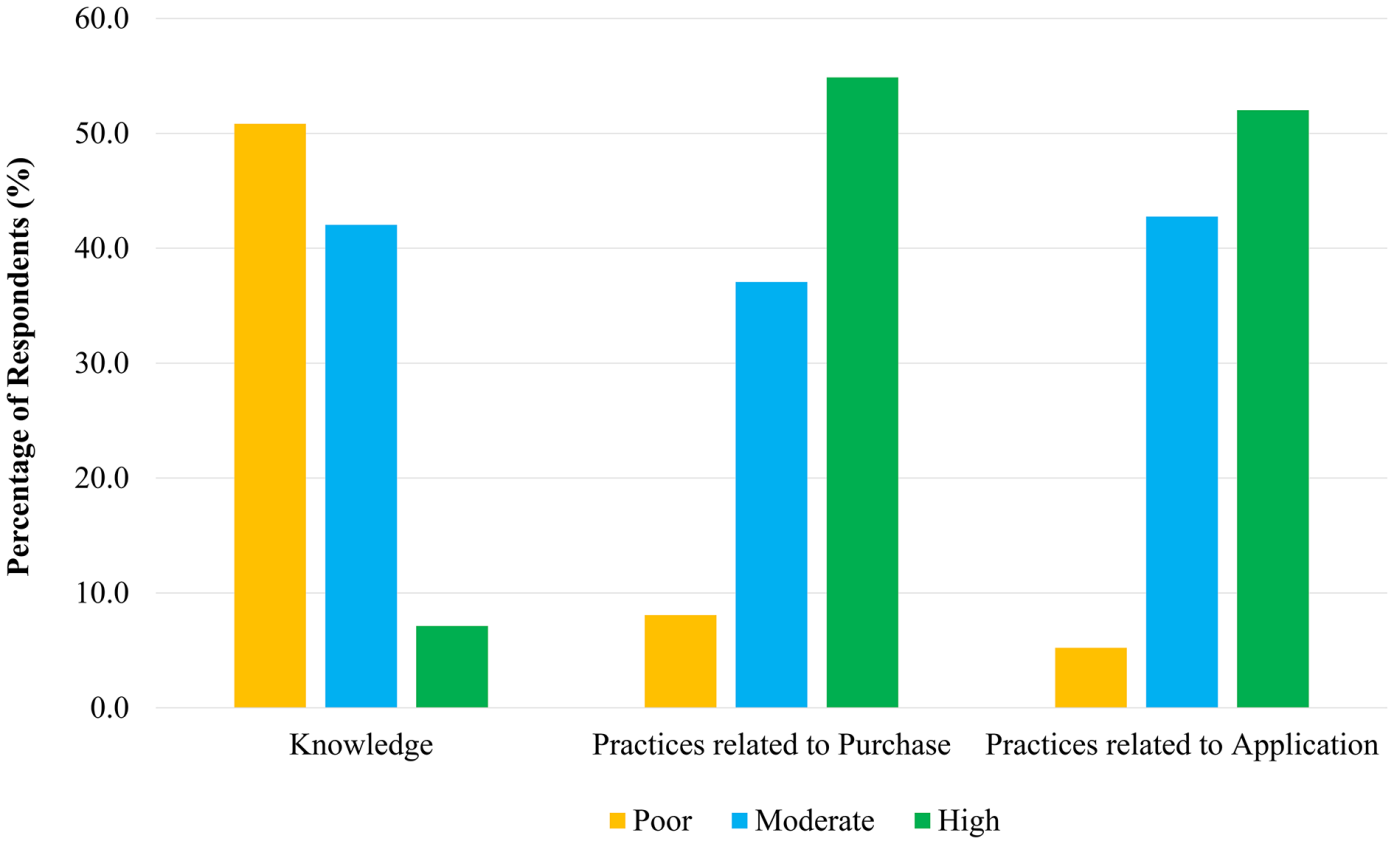
Distribution of the knowledge on cosmetics and practices related to purchase and application of cosmetics by the undergraduates.

During application of cosmetics, majority of the undergraduates always adhered to precautionary practices such as daily washing off the applied products (71.9%), preventing contamination of the products (66.9%), properly storing the products as indicated on the label of the product (62.2%), performing patch /sensitivity test before using (59.9%) and specifying products that you need before purchase (59.2%). However, it was noted that a considerable fraction of undergraduates was sharing cosmetics with friends (32.4%) and selecting available alternatives in case of unavailability of required product (43.7%), frequently, as shown in Table 4. Based on the overall practices related to purchase of cosmetics, around 54.9% of the undergraduates denoted a higher level of good practices during purchase of cosmetics, followed by undergraduates with a moderate level (37.1%) of practices. In case of practices related to application of cosmetics, only 52% of the respondents were characterized with a higher level of good practices, followed by 42.8% of undergraduates with a moderate level of good practices (Figure 1).

### Knowledge on cosmetic consumption

Of the respondents, 47.5% (*n* = 200) were characterized with a low level of knowledge on cosmetics, while around 43.9% (*n* = 185) were having a moderate knowledge. Therefore, only 8.6% of the undergraduates were having a high level of knowledge on cosmetics. However, a notable fraction of the students was aware of the harmful substances present in different cosmetic products and their potential adverse effects on humans as shown in Table 5. When the overall knowledge of undergraduates on cosmetics is considered, around 50.8% had a poor knowledge, while only 7.1% had a higher knowledge level (Figure 1).

**Table 5:** Knowledge on cosmetics.

Statement	Correct		Incorrect	
	*n*	%	*n*	%
The most common side effect of cosmetics on skin is acne.	175	41.6	246	58.4
Appropriate skin for using mint based cosmetic products is oily skin with acne	264	62.7	157	37.3
Oil based cosmetics have the most side effects on skin.	301	71.5	120	28.5
Yellowish discoloration of nails is the most common side effects of nail polish.	262	62.2	159	37.8
Antioxidant compounds in cosmetics hinder the production of melanin.	302	71.7	119	28.3
Cortisone is a common ingredient in skin whitening products.	348	82.7	73	17.3
Water proof mascaras are not safer for eye lashes.	312	74.1	109	25.9
Perfumes are a common prevalence cause for skin allergy.	306	72.7	115	27.3
Glutathione reduces oxidative stress/cell damage.	317	75.3	104	24.7
Vitamin C is an antioxidant ingredient in cosmetics, which prevent dark spots.	204	48.5	217	51.5
Lipsticks can contain heavy metals.	250	59.4	171	40.6

*n*, number of respondents in the sample.

### Risk factors for facing adverse health effects during cosmetic use

As suggested by the results of BLR, gender, academic year, knowledge on cosmetics, monthly expenditure on cosmetics, source of recommendation for cosmetics, practices related to purchase of cosmetics and consumption of cosmetics, preference to receive medical care in case of cosmetic related emergency, denoted significant associations with the incidence of adverse health effects due to cosmetic use (Table 6). The male undergraduates were reporting a significantly lower probability of facing cosmetic usage related health issues (*p* < 0.01; OR = 0.04; CI = 0.02–0.13) than the females. The highest prevalence rates of cosmetic usage related adverse health effects were observed among the undergraduates in the third academic year (96.2%, OR = 3.77), followed by fourth year (74.7%; OR = 1.42) and second year students (73.1%; OR = 1.39), when compared to first year students. Undergraduates with a higher knowledge on cosmetics (36.7%; OR = 0.01; CI = 0.00–0.04) reported the significantly least susceptibility to cosmetic related adverse health effects (*p* = 0.02), compared to undergraduates with a poor knowledge level (Table 6). Spending of a higher amount of money for cosmetics also denoted a significantly positive association with the incidence of cosmetic related adverse health effects (*p* = 0.01). The highest susceptibility was observed from the undergraduates that spent >5,000 LKR per month on cosmetics (95.7%, OR = 3.59; CI = 1.99–4.93), when compared to undergraduates that spent <1,000 LKR.

**Table 6:** Results of the binary logistic regression analysis.

Parameter		Total number of respondents	Incidence of cosmetic related adverse effects		*p* value	Odds ratio (OR)	95% C.I. for OR	
			No	Yes			Lower	Upper
Gender	Female	330	13.9	86.1	<0.01	**Reference**		
	Male	91	63.7	36.3		0.04	0.02	0.13
Academic year	First year	136	39.0	61.0	0.01	**Reference**		
	Second year	104	26.9	73.1		1.39	1.11	1.69
	Third year	106	3.8	96.2		3.77	1.71	4.06
	Fourth year	75	25.3	74.7		1.42	1.16	1.90
Knowledge on cosmetics	Poor	214	4.2	95.8	0.02	**Reference**		
	Moderate	177	42.9	57.1		0.04	0.01	0.11
	High	30	63.3	36.7		0.01	0.00	0.04
Monthly expenditure on cosmetics (LKR)	<1,000	250	31.6	68.4	0.01	**Reference**		
	1,000–2,500	128	18.0	82.0		2.66	1.45	3.24
	2,500–5,000	20	5.0	95.0		3.35	1.67	4.73
	>5,000	23	4.3	95.7		3.59	1.99	4.93
Source of recommendation	Medical Officers	44	43.2	56.8	0.04	**Reference**		
	Beauticians/ Pharmacists	100	35.0	65.0		1.41	1.12	2.14
	Friends	164	11.6	88.4		3.80	2.30	4.56
	Parents	53	41.5	58.5		1.07	1.01	1.88
	Elder Siblings	37	10.8	89.2		3.97	2.71	4.67
	Fiancé/Fiancée/Spouse	23	21.7	78.3		2.74	3.89	1.88
Practices related to purchase of cosmetics	Poor	34	2.9	97.1	0.03	**Reference**		
	Moderate	156	19.9	80.1		0.32	0.12	0.61
	High	231	31.2	68.8		0.17	0.08	0.37
Practices related to consumption of cosmetics	Poor	22	27.3	72.7	0.01	**Reference**		
	Moderate	180	11.7	88.3		0.84	0.55	0.95
	High	219	35.2	64.8		0.51	0.38	0.76
Do you receive medical care in case of cosmetic related emergency?	No	313	17.9	82.1	0.01	**Reference**		
	Yes	108	44.4	55.6		0.28	0.11	0.70

*p*-value, probability value; C.I., confidence interval; LKR, Sri Lankan rupees.

Interestingly, undergraduates that seek the advice from elder siblings (89.2%; OR = 3.97; CI = 2.71–4.67), friends (88.4%; OR = 3.80; CI = 2.30–4.56) and fiancé/fiancée/spouse (78.3%; OR = 2.74; CI = 3.89–1.88) to select cosmetics denoted a significantly (*p* = 0.04) higher tendency to have adverse health effects, compared to students that consulted medical officers (Table 6). Meanwhile, students that seek medical attention at the onset of any cosmetic related emergency showed a significantly lower susceptibility (*p* = 0.01; OR = 0.28; CI = 0.11–0.70). In addition, undergraduates that had a higher level of cosmetic purchase (*p* = 0.03; OR = 0.17; CI = 0.08–0.37) and application (*p* = 0.01; OR = 0.51; CI = 0.38–0.76) related practices showed a significantly lower prevalence level of adverse health, as shown in Table 6.

## Discussion

Cosmetics are materials or substances, which are being used to enhance the appearance of human body (7, 10, 13). The history of cosmetics dates back to ancient Egypt, where in the modern world consumers have different motives and drivers leading to diverse consumption patterns of cosmetics, to maintain or elevate their self-image (27). According to the outcomes of the present study, the majority (96.4%) of the studied population were using at least one type of cosmetic item, while the most prevalently used cosmetics product was perfume (65.6%), followed by face cream (63.2%) and body lotion (60.6%). In a similar study conducted by Getachew and Tewelde (28) at the Wollo University, North-east Ethiopia has reported a similar trend, where most of the students (97.3%) have the habit of using one or more cosmetic products. Further, lipstick, body lotions and eye makeup have been recognized as the most used products (28). Few recent studies conducted in the region have found perfume/deodorant, kajal, mascara and complexion lightening cream as widely used cosmetic products (8, 29).

The current study disclosed that more than half of the respondents were spending less than LKR 1000 (<3.50 USD, as of April 2022) on a monthly basis for cosmetics, while a relatively smaller fraction (10.3%) was spending more than LKR 2500 (>8.3 USD) per month. A recent study by Shiraz and Rahaman (29) has reported that female students in India were spending an average of 1,000 Indian Rupees (13.1 USD), while in another study conducted by Guo (30) males in Helsinki region had reported a monthly expenditure of € 22.1 (24 USD) for cosmetics and personal care products. Further, it was interesting to note that in Saudi Arabia the monthly expenditure on whitening and bleaching products has ranged from 60 to 100 USD (31). However previous studies have emphasized the fact that personal monthly income and usage of cosmetics are positively correlated (8, 32).

Regarding the usage frequency, cosmetics were mostly consumed routinely on a daily basis, whereas 77.0% of the undergraduates were using 1 to 2 cosmetic products at once, which varied up to 3 to 4 products (19.2%). Several studies have reported similar findings, where majority of the cosmetic users tend to utilize 1–3 cosmetic products routinely on a daily basis, with a frequency of twice to thrice per day (17–28). In another study by Alanzi et al. (32), a notable fraction of the respondents was using cosmetic items multiple times (more than thrice) per day, while bleaching products were used at least once daily. However, using multiple cosmetics at the same time, might increase the synergistic action of cosmetic products with the increased concentration of ingredients, leading to a higher probability of adverse health effects.

The usage of cosmetics is governed by different self-defined factors. Findings of the current study suggested that enhancing the appearance (89.3%), improving self-image/self-esteem (89.3%), being fashionable (67.9%) and protecting the skin against external environment (62.0%) are the major reasons behind the usage of cosmetics. Few recent studies have reported similar trends, where enhancing the appearance of the body and clothes, overall beatification, enhancement of style and enjoyment have emerged as major reasons behind cosmetic usage (8, 29–33). Cosmetic/beauty care shops (30.4%), pharmacies (26.7%) and supermarkets (20.0%) were the most preferred sources for purchase of cosmetic items. Several studies have reported that pharmacies are the most preferred choice for purchase of cosmetics in other countries (32–34). On the contrary, Kouotou et al. (10) has reported that majority of the Cameroonian female university students purchase cosmetic items, especially skin whitening items, via non-specialized stores and aestheticians.

Consumers tend to refer multiple information sources prior to use or purchase of cosmetics, in order to learn about the particular product or to compare a product with other competing brands (29, 31, 32). In this study, friends (39.0%) were found to be the dominant source of information for purchase of cosmetic products, followed by beauticians/ pharmacists (23.8%). Interestingly, only 10.5% of the undergraduates were seeking the advice of doctors in selecting cosmetic products for them. Many studies have revealed that the consumers are favoured in trying the products or brands that their friends or family members recommend, denoting that the word of mouth is highly influential in shaping the willingness to try and purchase cosmetic products (21, 35, 36). However, a study conducted in the Helsinki Region of Finland has evidenced that most of the males make their purchasing decisions by themselves, followed by the opinions of wife/fiancé and other male friends. Interestingly, the opinions of the medical practitioners or parents have not played a significant role in their cosmetic purchasing behaviour (30).

The current cosmetics industry utilizes a wide array of synthetic chemicals, including petrochemicals, parabens, sodium compounds, artificial colors, fragrance agents and preservatives. According to the previous studies, the negative effects on health caused by long-term exposure to such chemicals is apparent, where number of noxious or harmful mild to moderate effects related to cosmetic use have been reported. However, severe side effects are occasional (20, 29). The current study reported that majority of the undergraduates had experienced symptoms such as skin dryness (24.0%), acne (21%), allergies (20.5%) and rashes (19.8%) as adverse effects due to cosmetic usage. However, issues such as changes in skin colour, skin wounds, skin pigmentation and white heads were not prominent among the current study group. Many research studies have reported that, majority of the cosmetics users tend to have experienced at least one adverse health effect, mostly on face and hair, due to cosmetic products such as skin lightening creams, face/body lotions, sunscreen lotions, hair cosmetics, perfumes, lipsticks, mascara, and deodorants (8, 20, 28, 29). Findings of the present study were found to be consistent with several studies, where acne, loss of hair, body rashes, damage of nails, dermatitis and eczema, itching, skin dryness, sores on the scalp, redness of the eye and lip crack have been reported as the mostly manifested adverse effects (8, 28, 29, 34). In contrast, severe facial redness, wounds due to itching and scratching have been reported as least common adverse effects (21).

A relatively higher fraction (43.2%) of respondents of the present study had reacted to such adverse effects by stopping the use of suspected cosmetic products, while only 34.4% had pursued medical attention and 7.1% shifted the brand of the suspected cosmetics. Anyway, the respondents had paid limited attention on complaining regarding the problems that occur when using a relevant product to responsible authorities. A similar trend has been reported by several previous studies (8, 29, 36), where some cosmetic users simply ignore the side effects, without consulting medical officers or filing proper complains (34).

### Preferred product characteristics of cosmetics

Behavioural intention is theorized as an immediate origination of behaviour, which is resulted as a combination of attitudes and perceptions. The perception towards cosmetics and self-identity are correlated where the perception is influenced by personal experience, social networks, and marketing techniques (35). The attitudes of a consumer towards purchasing of cosmetics is often affected by different marketing strategies, along with product attributes such as brand, functions, ingredients, packaging, fragrance, and price (10, 21). Results of this study revealed that the majority pay attention to the ingredients of the cosmetic product (75.5%), while focusing on the convenience in application (68.4%), brand (68.7%), medical recommendations (65.5%) and endorsement by professionals or experts (62.5%) prior to purchase of any cosmetic product. In contrast, a previous study conducted in United Kingdom has revealed that around 25% of British women pay less attention on the ingredients in cosmetics, while the majority focus more on product functions. In terms of the brand, most of the consumers mind the brand name, but pay less attention on the original brand logo and place of manufacture to distinguish the authenticity of the cosmetic items they purchase (35).

Even though, product marketing and advertising play significant roles in popularizing cosmetics and beauty care products, the studied population had a limited attention on such advertising tools (25). Few previous studies also have shown that a fraction of consumers tend to exhibit a limited reliance on marketing tools or declarations of producers (10, 30). On the contrary, Dimitrova et al. (37) and Shrestha and Shakya (29) have indicated that, cognition of the cosmetic consumers could be altered and influenced by product marketing, quality attributes, price and delivery. Packaging and labeling also shared an equal importance in selection of cosmetics products to purchase, due to convenience of information and capability of attracting the consumers. The label provides key information on the product, such as ingredients, brand, adherence to quality regulations, product grading and instructions for usage (35).

Interestingly, most of the undergraduates were paying attention to expiry and manufacture dates (56.5%), usage instructions (45.4%) and the ingredients (40.4%) during purchase of cosmetic products. Comparatively, a lower level of attention was placed on manufacturing company (47.5%), brand credibility (41.1%) and quality certifications (40.1%), while ignoring the price of the product at the decision-making point of purchase, along with the production license of the manufacturing company. Several recent studies have reported similar findings, where reading user instructions, brand names, content, expiry dates and special remarks have been recognized as the most prevailing practices among cosmetic users (28, 29). As emphasized by Leclerc et al. (38), the brand name has a strong hedonic association with the purchasing intention, leading consumers to repeatedly purchase the same brand or to switch among several brands, due to the tangible quality of the product (2, 21, 28, 29). In addition to that, Rybowska (34) has reported that around 22% of the respondents never pay attention to the certifications or information on the packaging, while 34% respondents only notice this information accidentally. However, according to Cervellon et al. (39), consumers show a less tendency to purchase products without proper labeling.

Majority (71.9%) of the study population used to wash off the applied cosmetics daily, which can prevent the adverse health effects due to prolonged usage. Even though, performing of a patch test for cosmetics could avoid the potential of allergic reaction before usage, only 59.9% were performing a patch test. Few recent studies have reported even lesser rates of performing patch testing for allergy prior to use of cosmetics or personal care products (10, 28). Identifying the cosmetic requirements before purchase, avoids buying of unnecessary items, which may result accumulation of unused products and been thrown away without using (21). However, a considerable fraction (59.2%) of undergraduates of this study were used to specify products that they need to purchase (59.2%), whereas 43.7% of the respondents used to switch to any feasible alternative in case of unavailability of the required product. A similar trend was indicated by Kouotou et al. (10), where more than 40% of the respondents were satisfied with alternative products over the predetermined ones, in case of unavailability.

In general, nearly half of the study population were characterized with a poor level of knowledge on cosmetics, while a study conducted in Saudi Arabia has also reported a poor level of knowledge on the adverse effects of cosmetic usage among undergraduates (17). A previous study conducted in India has reported that even though the majority of study participants were showing a satisfactory knowledge on good practices regarding the cosmetic use, a notable portion was using the same brand of cosmetics regardless of suffering from adverse effects (8). Especially, the practices on the selection and application of cosmetic products have remained at an unfavourable level among females in Saudi Arabia, which could lead into higher prevalence rates of adverse health effects (20).

### Risk factors behind experiencing adverse health effects

The current study showed that gender, academic year, knowledge on cosmetics, monthly expenditure on cosmetics, source of recommendation for cosmetics, practices related to purchase of cosmetics and consumption of cosmetics, preference to receive medical care in case of cosmetic related emergency, delineate significant associations with the incidence of adverse health effects due to cosmetic usage. The female undergraduates reported a significantly higher probability of facing cosmetic usage related health issues, compared to their male counterpats. Extensive and prolonged usage rates of cosmetics and beauty care products and more attention placed on their appearance by females could be the major reasons for this trend (20, 40). A previous study conducted in Sri Lanka has reported a significant association among the incidence of facial acne and frequent exposure to cosmetic products in adolescent females (4). Meanwhile, the highest prevalence rates of cosmetic usage related adverse health effects were observed among the undergraduates in the third academic year, followed by fourth year. Few previous studies conducted in the region have shown a positive association among the academic year of undergraduates and self-medication behaviour (41–43).

The lack of knowledge of the undergraduates regarding the harmful substances in cosmetics and their adverse health effects caused by the extensive and prolonged usage, have been identified as a key reason behind the prevalence of adverse health effects (17, 29). Findings of the current study also advocated a similar trend, where undergraduates with a poor knowledge on cosmetics reported the significantly highest susceptibility to cosmetic related adverse health effects. Further, undergraduates that had a poor level of good practices related to purchase and application of cosmetic products reported significantly higher incidence levels of adverse health effects, which agrees with the findings of previous studies (20, 40). Undergraduates with a relatively higher monthly expenditure on cosmetics denoted a significantly higher likelihood for suffering from cosmetic related adverse health effects. A similar trend has been reported in the Jimma University of Southwest Ethiopia. However, the age, educational level or the source of purchase have not shown any positive associations with the incidence of adverse health effects (28).

Undergraduates that select their cosmetic products based on the advice of elder siblings, friends or fiancé/fiancée/spouse, denoted a significantly higher probability to suffer from adverse health effects compared to students that consulted medical officers or professional beauticians. Often, elder siblings, friends or fiancé/fiancée/spouse lack an in-depth knowledge on cosmetics and provide their recommendation based on their experience, which could be the underlying reason for this observation (40). Meanwhile, students that seek medical attention at the onset of any cosmetic related emergency reported a significantly lower level of susceptibility to cosmetic related adverse health effects. Consultation of qualified dermatologists could enable the patients to treat their health effects at the initial stages, without allowing those to adverse and to select appropriate cosmetics suitable for their condition (15, 40).

### Limitations of the study

The current study considered undergraduates belonging to five major universities of Sri Lanka, out of the 15 government universities in Sri Lanka, which remains as a limitation. However, a satisfactory proportion of undergraduate community was recruited for the current study, representing all the academic disciplines to compensate for aforementioned limitation. In addition, a recall bias could be expected since the cosmetic usage practices were reported by the undergraduates, which remains a general limitation in this type of studies (43).

## Conclusions and recommendations

Current study reveals a higher cometic usage rate among the undergraduates, along with a higher prevalence rate of cosmetic related adverse health effects. This should be considered as a critical factor in health care management, since cosmetic related health effects are often overlooked in many developing countries. Limited preference towards logging complains regarding such issues denoted by the studied population also aggravates this situation. This warrants the necessity of more effective monitoring and supervision systems to regulate the cosmetics market in Sri Lanka.

Gender, academic year, knowledge on cosmetics, monthly expenditure on cosmetics, source of recommendation for cosmetics, practices related to purchase of cosmetics and consumption of cosmetics and preference to receive medical care in case of cosmetic related emergency were recognized as the significant risk factors associated with the incidence of cosmetic related adverse health effects among the undergraduate students. As highlighted by the current study more awareness on the adverse and chronic effects of cosmetics, harmful ingredients to be avoided and safe practices for purchase and use of cosmetics is essential. Therefore, conducting health education programmes on safe cosmetic usage, is imperative for the adolescents. These programmes should be tailored to address the societal and cultural needs of the local settings. In addition, effective tools and regulations are recommended to be implemented, to promote responsible cosmetic usage within Sri Lanka.

## Data availability statement

The raw data supporting the conclusions of this article will be made available by the authors, without undue reservation.

## Ethics statement

The studies involving human participants were reviewed and approved by Ethics Review Committee (ERC) of the Faculty of Agriculture & Plantation Management, Wayamba University of Sri Lanka. Written informed consent for participation was not required for this study in accordance with the national legislation and the institutional requirements.

## Author contributions

LU, NS, and MU: conceptualization and methodology. NS and PS: data curation. LU: formal analysis. LU and NS: investigation and Writing – original draft. MU: writing – review and editing. TR: review and editing. All authors contributed to the article and approved the submitted version.
